# Accelerating 3D single-molecule localization microscopy using blind sparse inpainting

**DOI:** 10.1117/1.JBO.26.2.026501

**Published:** 2021-02-27

**Authors:** Sunil Kumar Gaire, Yanhua Wang, Hao F. Zhang, Dong Liang, Leslie Ying

**Affiliations:** a The State University of New York at Buffalo, Department of Electrical Engineering, Buffalo, New York, United States; b Beijing Institute of Technology, School of Information and Electronics, Beijing, China; c Northwestern University, Department of Biomedical Engineering, Evanston, Illinois, United States; d Chinese Academy of Sciences, Shenzhen Institute of Advanced Technology, Paul C. Lauterbur Research Center for Biomedical Imaging, Shenzhen, Guangdong, China; e The State University of New York at Buffalo, Department of Biomedical Engineering, Buffalo, New York, United States

**Keywords:** single-molecule localization microscopy, super-resolution, 3D imaging, inpainting, microtubules, image reconstruction, optimization

## Abstract

**Significance:** Single-molecule localization-based super-resolution microscopy has enabled the imaging of microscopic objects beyond the diffraction limit. However, this technique is limited by the requirements of imaging an extremely large number of frames of biological samples to generate a super-resolution image, thus requiring a longer acquisition time. Additionally, the processing of such a large image sequence leads to longer data processing time. Therefore, accelerating image acquisition and processing in single-molecule localization microscopy (SMLM) has been of perennial interest.

**Aim:** To accelerate three-dimensional (3D) SMLM imaging by leveraging a computational approach without compromising the resolution.

**Approach:** We used blind sparse inpainting to reconstruct high-density 3D images from low-density ones. The low-density images are generated using much fewer frames than usually needed, thus requiring a shorter acquisition and processing time. Therefore, our technique will accelerate 3D SMLM without changing the existing standard SMLM hardware system and labeling protocol.

**Results:** The performance of the blind sparse inpainting was evaluated on both simulation and experimental datasets. Superior reconstruction results of 3D SMLM images using up to 10-fold fewer frames in simulation and up to 50-fold fewer frames in experimental data were achieved.

**Conclusions:** We demonstrate the feasibility of fast 3D SMLM imaging leveraging a computational approach to reduce the number of acquired frames. We anticipate our technique will enable future real-time live-cell 3D imaging to investigate complex nanoscopic biological structures and their functions.

## 1 Introduction

Single-molecule localization microscopy (SMLM) such as (direct) stochastic optical reconstruction microscopy [(d)STORM],^1^^,^^2^ (fluorescence) photoactivated localization microscopy [(f)PALM],^3^^,^^4^ and other variants^5^^–^^8^ have extended the imaging resolution of conventional optical fluorescence microscopy beyond the diffraction limit (∼250  nm). In these methods, a random and sparse subset of fluorophores in the sample is imaged in each diffraction-limited image frame, whereas a large number of such frames are obtained sequentially. Then, the detected individual fluorophores in each frame are precisely localized, and finally, all the localization positions from these frames are assembled together to generate the super-resolution image. Three-dimensional (3D) SMLM^9^^–^^16^ requires additional axial (z axis) information, which is obtained by using z dependent point spread function (PSF).^17^ Optically engineered PSFs such as astigmatic,^9^ double-helix,^18^ biplane,^19^ interferometric,^20^ airy-beam,^21^ and tetrapod^12^ are commonly used in existing 3D SMLM imaging to encode the axial information of blinking fluorescent molecules. PSFs shapes are generally engineered via the introduction of optical elements such as cylindrical lens,^9^ phase mask,^22^ or deformable mirror^15^ in the imaging pathway of the microscope. In both 2D and 3D SMLM imaging, to achieve sufficient dense localizations to reveal biological samples’ details, a large number of sequential diffraction-limited frames (typically $>10^{4}$) are needed, suggesting a long acquisition time. Such slow imaging makes potential live-cell and high-throughput imaging more challenging. Practically, the acquisition of such long frame sequences also results in the degradation of image quality due to the dyes’ photobleaching. Furthermore, the processing of such a large number of image frames requires considerable processing times.^23^ Therefore, a faster SMLM technique is always desirable.

Several approaches have been reported to accelerate the imaging and data processing time of SMLM. One of them is to increase the fluorophore blinking kinetics using a high-power laser and to use a high-speed camera (with higher frames per second) to capture those fast blinking single-molecule events.^10^^,^^24^ Huang et al.^25^ achieved video-rate SMLM using scientific complementary metal-oxide-semiconductor (sCMOS) cameras. These acceleration methods provide faster imaging at the cost of image quality degradation.^10^^,^^26^ Specifically, high-excitation laser intensity and fast detection decreased the photon count per localization, resulting in deterioration of localization precision and resolution.^26^ Another approach is to increase the number of active fluorophores per frame.^27^^,^^28^ However, the high activation density causes fluorescent spots to overlap in the diffraction-limited images, making it more difficult to precisely localize the fluorophores.^28^ Despite this challenge, most of the existing techniques^29^^–^^31^ use higher molecular density per frame to increase the imaging speed. Recently, deep learning has been used to accelerate the SMLM methods. Typically, deep learning is implemented to precisely localize the 2D or 3D position (or color separation in case of multicolor imaging) of blinking single-molecules PSFs in each frame.^32^^–^^39^ These methods ultimately accelerate the data processing time of SMLM methods, but still require a large number of frames. Further, deep learning is leveraged by Ouyang et al.^40^ to accelerate 2D SMLM and by Gaire et al.^41^ to accelerate 2D multicolor spectroscopic SMLM using very few frames. However, the limitation of a deep learning method is that it requires a large quantity of training data with similar structures.

Here, we present a computational approach to accelerate 3D SMLM imaging. The experimental setup, data acquisition procedure, and localization methods remain the same as those of standard 3D SMLM methods, except that very few diffraction-limited frames are acquired, which will reduce the acquisition time and ultimately accelerate imaging speed. Further, the data processing time will also be reduced accordingly. For the standard 3D SMLM method, the final image rendered from very few frames is sparse and provides less information to extract the biological sample’s fine structures. Our approach can recover those unresolved structures in the sparse image with low emitter densities and reconstruct the high-quality 3D super-resolution image. The high-density estimation of 2D SMLM imaging using the blind sparse inpainting has been previously reported in detail.^42^ Here, we extended it to accelerate 3D SMLM imaging by introducing a sparsifying transform appropriate for the 3D structure. In our previous work, high-density 2D SMLM images were reconstructed by solving an $l_{1}$ minimization problem using the alternating direction method of multipliers (ADMM)^43^ with curvelet transform^44^ as the sparsifying transform. Here, we also use ADMM but with combined curvelet transform and an additional total variation (TV) regularization for the depth direction. We confirm the efficacy of the proposed algorithm using both simulated and experimental 3D SMLM datasets. The preliminary results of this article were reported in Ref. 45. This expanded article includes additional simulation, experimental, and quantitative evaluation results and their analysis.

## 2 Reconstruction Approach

**Fig. 1:**
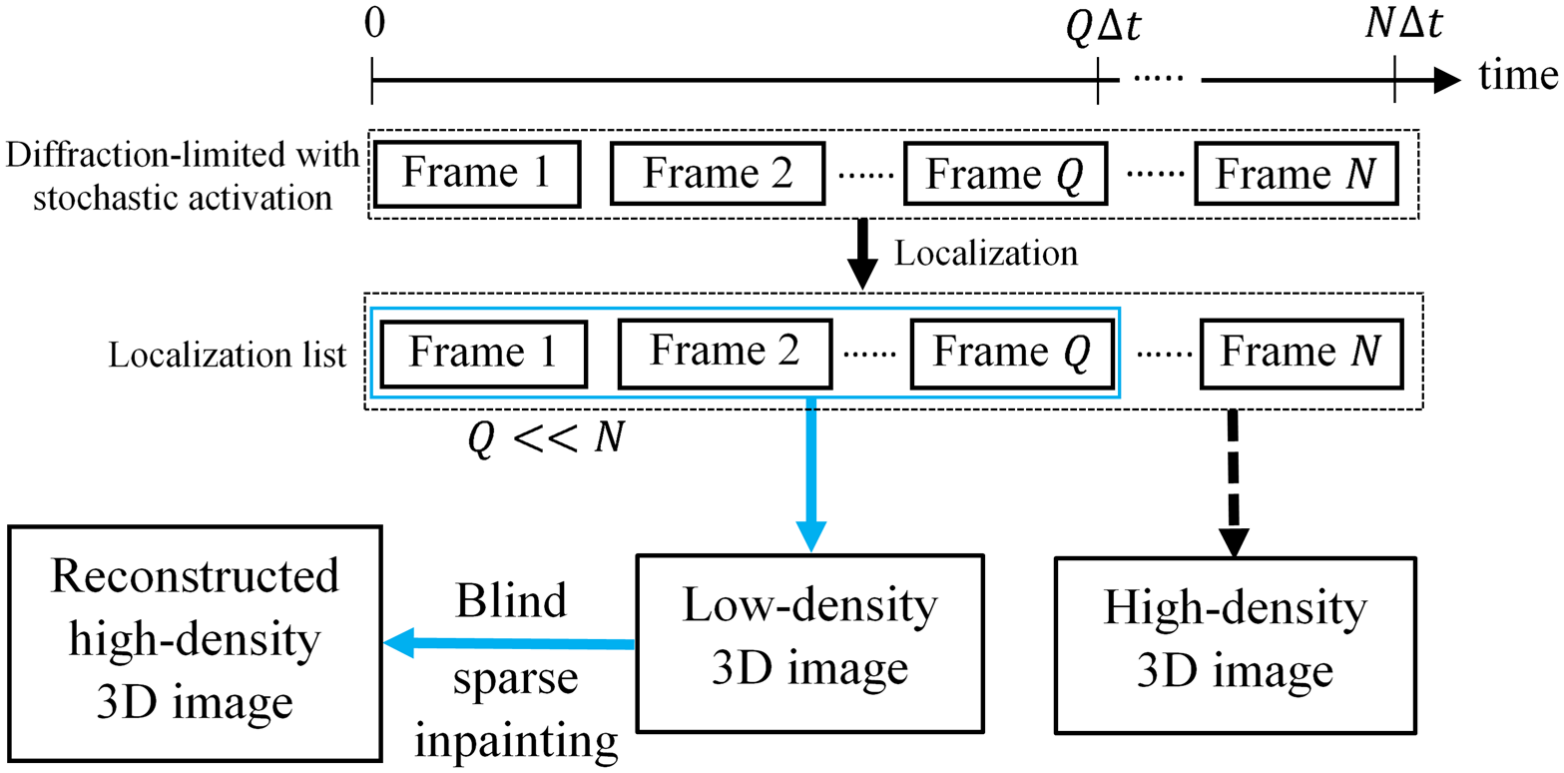
Comparison of blind sparse inpainting method with the existing 3D SMLM method. 3D super-resolution image in standard SMLM is obtained by imaging and processing a large number of diffraction-limited single-molecule frames (suppose N frames). The proposed method uses very few diffraction-limited frames (suppose Q frames and Q≪N), which results in a low-density 3D image. The high-density 3D image is then reconstructed using blind sparse inpainting.

In standard 3D SMLM, a large number of diffraction-limited frames (suppose N frames) are imaged with a total acquisition time of NΔt, where Δt is the time to acquire a single frame (typically 10 to 30 ms) and processed to produce a high-density 3D super-resolution image. A smaller number of frames (suppose Q frames and Q≪N) with a very short acquisition time of QΔt will generate a low-density 3D image (Fig. 1). Our goal is to reduce the acquisition time by reconstructing the high-density 3D super-resolution image using a low-density 3D image acquired using fewer frames, which is sparse and incomplete. For reconstruction, we need to restore the unknown fluorophore localization positions based on the available fluorophore localization points on the low-density 3D image. Thus, the restoration problem can be formulated as an image inpainting task aiming to restore the mission regions of the corrupted image and reconstruct the original image.

Mathematically, the relationship between the vectorized low-density 3D image $x_{Q}$ from the localization emitters acquired in Q frames and the desired high-density 3D image vector x can be modeled as

$$x_{Q}=P_{Q}x,$$ (1)

where $P_{Q}$ is a diagonal matrix with either element 1, for the acquired location or 0, for the missing location. To solve Eq. (1), we first need to estimate the unknown measurement matrix $P_{Q}$ (called “blind”) based on the low-density 3D image and then reconstruct x from $x_{Q}$. The estimation of $P_{Q}$ is challenging in the sense that a zero-valued pixel in $x_{Q}$ can be background without any fluorophore or those with fluorophore but not detected in the acquired Q frames. The locations of fluorescence molecules captured in Q frames are determined by performing hard-thresholding on the low-density image $x_{Q}$.

After $P_{Q}$ is obtained, x can be estimated from $x_{Q}$, which is still nontrivial because of infinite possible solutions. Prior information has to be exploited as a constraint to obtain a unique reconstruction with good fidelity to the true structures. Here, we reconstruct the desired high-density 3D image by employing sparseness as an image prior. Specifically, the high-density 3D image is reconstructed by solving the following unconstrained minimization problem

$$\underset{x}{\min}\;\lambda_{1}{\left\|P_{Q}x-x_{Q}\right\|}_{2}^{2}+{\left\|\Phi x\right\|}_{1}+\lambda_{2}\mathrm{TV}(x),$$ (2)

where ${\left\|\cdot \right\|}_{1}$ and ${\left\|\cdot \right\|}_{2}$ represent $\ell_{1}$ and $\ell_{2}$ norms, respectively, $\lambda_{1}$ and $\lambda_{2}$ are the weight parameter and regularization parameter, respectively, Φ represents a sparsifying transform, and TV(·) is a total variation regularization. The first term enforces data consistency, the second term enforces the sparsity in the transform domain, and the third term promotes the piecewise smoothness of the image.

The choice of sparsifying transform depends on the image content and plays a crucial role in image reconstruction. Many biological structures, such as microtubules, are of anisotropic curve-like nature. Therefore, we use the curvelet transform as a sparsifying transform in the lateral plane. It provides sparsity and excellent directional sensitivity and anisotropy. Thus, curvelet transform can efficiently characterize anisotropic features such as curves, edges, and arcs.^46^ The discrete curvelet transform was implemented using CurveLab^47^ with curvelets via wrapping approach. It includes four steps: 2D fast Fourier transform (FFT) , windowing, frequency wrapping, and 2D inverse FFT.^44^ TV regularization is used in the depth direction only. TV is defined as $\mathrm{TV}(x)={\left\|\mathrm{Gx}\right\|}_{1}$, where G is the first-order finite-difference operator along the depth direction and ${\left\|\cdot \right\|}_{1}$ denotes $\ell_{1}$ norm. More detail about the optimization algorithm is in the next section.

## 3 Optimization Algorithm

The convex optimization problem of Eq. (2) is a standard $\ell_{1}$ minimization problem. It can be solved using efficient approaches such as variable splitting and augmented Lagrangian method (ALM).^48^^,^^49^ In this paper, we are using a specific variation of ALM called ADMM.^43^ We first introduce the auxiliary variable d=Φx and e=Gx in Eq. (2) to decouple the $\ell_{1}$ terms from other parts and obtain the following equivalent form

$$\underset{x}{\min}\;\lambda_{1}{\left\|P_{Q}x-x_{Q}\right\|}_{2}^{2}+{\left\|d\right\|}_{1}+\lambda_{2}{\left\|e\right\|}_{1}\;\mathrm{s.t.}\;\Phi x=d\;\text{and}\;\mathrm{Gx}=e.$$ (3)

The scaled form of the augmented Lagrangian function (ALF) of Eq. (3) can be written as

$$L(x,d,e,u,v)=\lambda_{1}{\left\|P_{Q}x-x_{Q}\right\|}_{2}^{2}+{\left\|d\right\|}_{1}+\lambda_{2}{\left\|e\right\|}_{1}\;+\frac{\rho }{2}{\left\|\Phi x-d+u\right\|}_{2}^{2}+\frac{\mu }{2}{\left\|\mathrm{Gx}-e+v\right\|}_{2}^{2},$$ (4)

where u and v are Lagrangian multipliers representing scaled dual variables. Similarly, ρ and μ are the penalty parameters. The ADMM iteration scheme will be

$$x^{k+1}=\arg\underset{x}{\min}\;\lambda_{1}{\left\|P_{Q}x-x_{Q}\right\|}_{2}^{2}+\frac{\rho }{2}{\left\|\Phi x-d^{k}+u^{k}\right\|}_{2}^{2}+\frac{\mu }{2}{\left\|\mathrm{Gx}-e^{k}+v^{k}\right\|}_{2}^{2},$$ (5)

$$d^{k+1}=\arg\underset{d}{\min}\;{\left\|d\right\|}_{1}+\frac{\rho }{2}{\left\|\Phi x^{k+1}-d+u^{k}\right\|}_{2}^{2},$$ (6)

$$e^{k+1}=\arg\underset{e}{\min}\;\lambda_{2}{\left\|e\right\|}_{1}+\frac{\mu }{2}{\left\|{\mathrm{Gx}}^{k+1}-e+v^{k}\right\|}_{2}^{2},$$ (7)

$$u^{k+1}=u^{k}+\Phi x^{k+1}-d^{k+1},$$ (8)

$$v^{k+1}=v^{k}+{\mathrm{Gx}}^{k+1}-e^{k+1}.$$ (9)

The x-subproblem has a closed-form solution

$$x^{k+1}=B(2\lambda_{1}P_{Q}^{T}x_{Q}+\rho \Phi^{H}(d^{k}-u^{k})+\mu G^{T}(e^{k}-v^{k})),$$ (10)

where $B={\left(2\lambda_{1}P_{Q}^{T}P_{Q}+\rho I+\mu I\right)}^{-1}$. The superscripts H and T denote the Hermitian transpose and the transpose of a matrix, respectively. The optimum values of d-subproblem and e-subproblem are obtained through the element-wise shrinkage operator^48^

$$d^{k+1}=shrink(\Phi x^{k+1}+u^{k},\frac{1}{\rho }),$$ (11)

$$e^{k+1}=shrink({\mathrm{Gx}}^{k+1}-v^{k},\frac{\lambda_{2}}{\mu }),$$ (12)

where shrink(.) is defined as

$$shrink(x,\gamma )=\frac{x}{\left|x\right|}\;\max(|x|-\gamma ,0).$$ (13)

The algorithm terminates when the predefined maximum number of iteration is reached. The proposed ADMM optimization algorithm for blind sparse inpainting is summarized in Algorithm 1. The algorithm was implemented in MATLAB^®^ R2018a.

**Algorithm 1:** 

**Input:**$x_{Q}$-low-density 3D image.
$\lambda_{1}$, $\lambda_{2}$-weight and TV regularization parameters.
ρ, μ-penalty parameters.
Φ-sparsifying transform operator.
n-maximum number of iterations (stopping criteria).
**Output:**x-high-density 3D image.
**Initialization:**$d^{0}=0$, $e^{0}=0$, $u^{0}=0$, $v^{0}=0$, count=1.
**for**count=1:n**do**
Solve x-subproblem using Eq. (10).
Solve d-subproblem using Eq. (11).
Solve e-subproblem using Eq. (12).
Update u using Eq. (8).
Update v using Eq. (9).
**End for**.

All the parameters in our implementation were tuned heuristically, and the best results obtained from the quantitative evaluations are presented. In general, the weight parameter $\lambda_{1}$ balances the sparsity constraint/smoothness and data consistency. Typically, smaller $\lambda_{1}$ weights the smoother image, while large $\lambda_{1}$ penalizes data consistency more (preserving more acquired information). Such control of sparsity constraint and data consistency in the lateral direction is also affected by the value of ρ. Similarly, the smoothness and data consistency in the axial direction is also controlled by the parameter μ. Due to the variation of intensity and density in each image, a single value of these parameters may not work for all images. To simplify the parameter-tuning process of all images, the maximum intensity was truncated to 255, and then intensity values were rescaled to the interval of [0, 1]. In our implementation, we used the value of $\lambda_{1}$ in the range of 10 to 80, and ρ in the range of 10 to 150. Similarly, $\lambda_{2}=10^{-6}$ and μ=0.1/0.01 were used. The results are insensitive to a small change in the values of these parameters.

## 4 Results

### 4.1 Simulation Results

To demonstrate the performance of blind sparse inpainting reconstruction, we used two sets of simulated localization data.

**Fig. 2:**
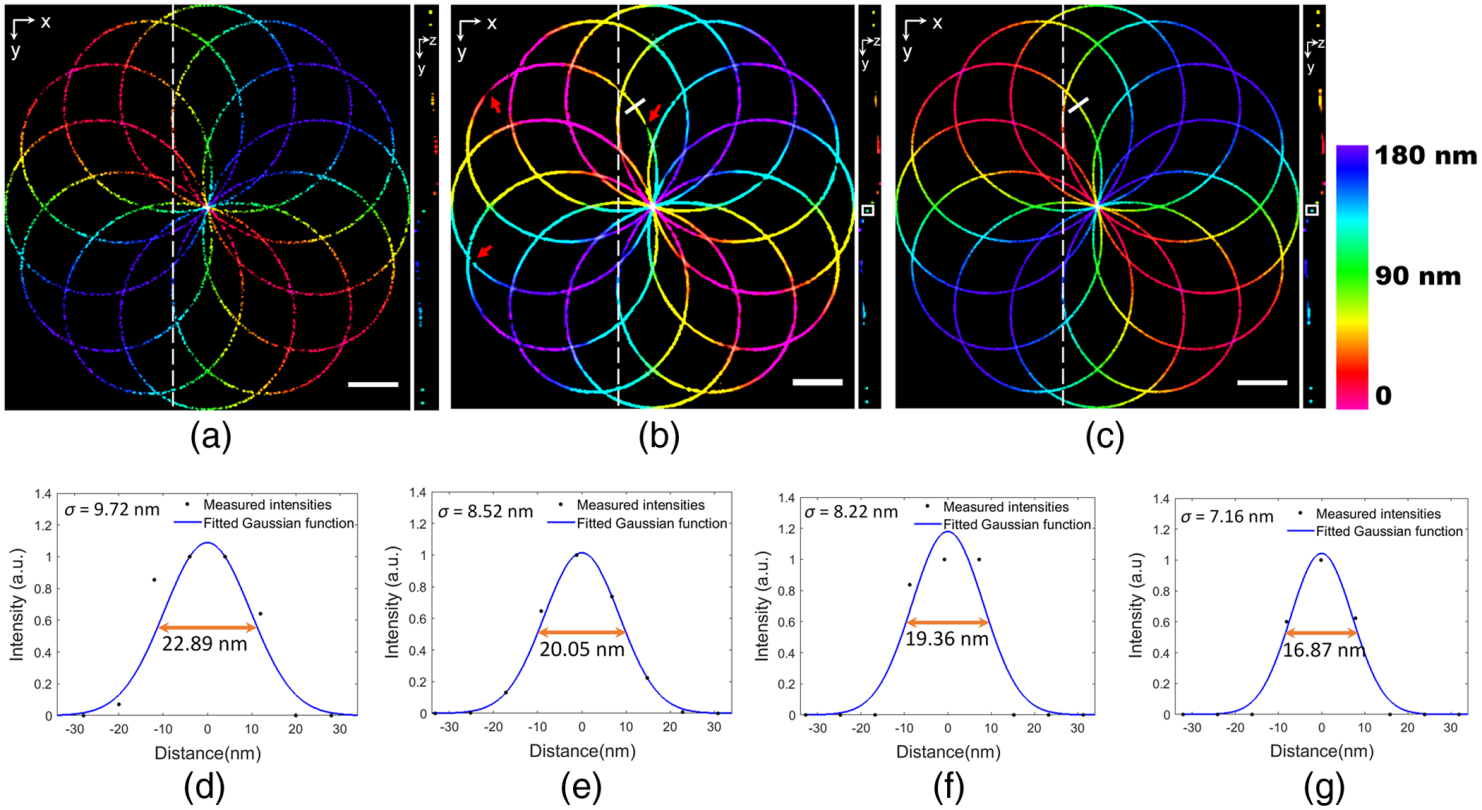
Blind sparse inpainting reconstruction of simulated 3D SMLM image. (a) Low-density image using 1000 frames; (b) blind inpainting reconstruction; and (c) high-density ground-truth image using 10,000 frames (also see Video 1 and Video 2). The right panel in each image shows a (y,z) slice at the position indicated by the white dashed line. The color bar shows the depth of z. Pixel size: 8 nm. Scale bars: 0.5  μm. (d) and (e) Intensity profile and FWHM at the white line segment shown in the (b) reconstructed image and (c) the ground-truth image, respectively. (f) and (g) Intensity profile and FWHM along the line segment (not shown) on z direction at white boxes on (y,z) slices in the images (b) and (c), respectively. Black dots in the intensity profiles are measured intensities, and blue curves are fitted Gaussian functions, with standard deviation σ and FWHM (double orange arrow) as indicated [Video 1, MP4, 5.5 MB [URL: https://doi.org/10.1117/1.JBO.26.2.026501.1]; Video 2, MP4, 4 MB https://doi.org/10.1117/1.JBO.26.2.026501.2].

For the first one, we generated a simulated 3D SMLM image in the shape of a knot as the “ground-truth” specimen. The knot had a volume of dimension $4.02\times 4.02\times 0.18\;\mu m^{3}$. The localization list was simulated by randomly selecting some locations in the knot to mimic the activated molecules with an activation density of approximately ten molecules per frame ($0.62\text{ molecules}/\mu m^{2}$ per frame).^16^ We directly recorded the localized coordinates (x,y,z) and their intensities of blinking molecules in each camera frame. Since the localization emitters were directly obtained from the true image, there were no localization errors or background noise. The localization list was then used to render the 3D image. The increasing density images can be synthesized by combining these localization points using an increasing number of frames. The resulting high-density super-resolution 3D image (Video 1) has lateral and axial resolutions of ∼20 and ∼17  nm, respectively [Figs. 2(e) and 2(g)]. We used fewer frames to generate the low-density 3D image and then applied our algorithm to reconstruct the high-density 3D image.

**Fig. 3:**
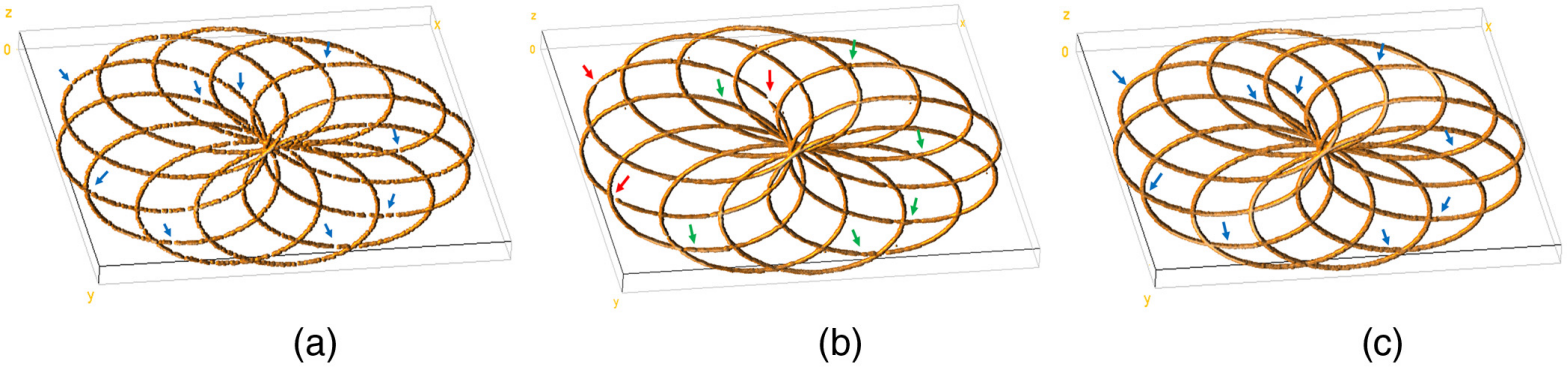
Volume visualization of the simulated 3D image. (a) Low-density; (b) reconstructed; and (c) ground-truth images, respectively. The low-density image was rendered using 1000 frames and the ground-truth image was obtained using 10,000 frames.

To reconstruct the high-density 3D image from the low-density image, we constructed 22 z-slices of the low-density 3D image by grouping the localization data in the z axis with thickness 8 nm. ThunderSTORM,^50^ an open-source SMLM data analysis plugin for Fiji,^51^ was used to computationally render the z-stack with the 3D simulated localization list as an input. Due to simultaneous reconstruction of multiple z-slices (lateral and axial direction), the reconstruction of the 3D SMLM image is much more complicated compared to the reconstruction of the 2D SMLM image as in Ref. 42. The result in Fig. 2(b) shows that the blind sparse inpainting reconstruction of the low-density image rendered with Q=1000 frames and having 15,910 localization points significantly improved the density and is visually equivalent to the ground-truth [Fig. 2(c)] rendered with N=10,000 frames with a total of 96,203 fluorophore localization points. The 3D projection of Figs. 2(a)–2(c) is presented in Video 2. Additionally, the volume visualization of the simulated low-density, blind-inpainting reconstruction, and ground-truth 3D images using the Volume Viewer^52^ plugin in Fiji is shown in Fig. 3. Most of the incomplete and rough curvilinear structures due to reduced localization points in the low-density image are reconstructed almost perfectly, giving complete and continuous filament structures with an excellent agreement with the ground-truth image. At some positions, where the input low-density image has very little information available, the reconstruction still deviates from the ground-truth [red arrows in Figs. 2(b) and 3(b)]. Such errors can be reduced by increasing the frame numbers (thereby the number of localization points), but at the cost of reduced acceleration.

**Fig. 4:**
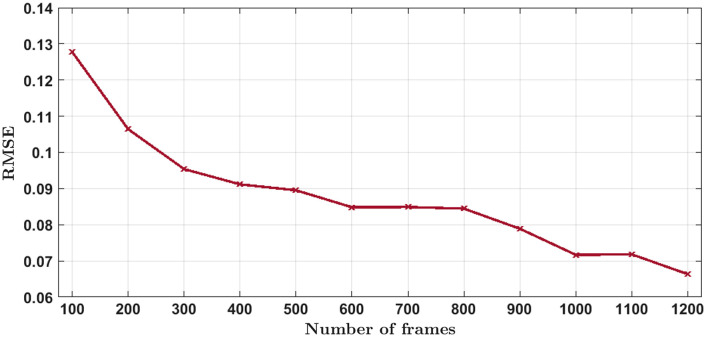
Quantitative evaluation of knot simulation results using RMSE for the different number of frames.

The reconstructed image resolution was evaluated using the full width at half maximum (FWHM) of the intensity profile. The FWHM values along the lateral and axial direction for the reconstructed image are shown in Figs. 2(d) and 2(f), respectively. Similarly, Figs. 2(e) and 2(g), respectively, show FWHM values of the ground-truth image in lateral and axial directions. The intensity profile in lateral direction was taken along the white line segments in Figs. 2(b) and 2(c). Similarly, line segments (not shown) along the z direction at the white boxes on (y,z) slice of Figs. 2(b) and 2(c) were used to obtain axial intensity profiles. The black dots in the intensity profiles are measured intensities, and the blue curves are fitted Gaussian functions, with standard deviation σ and FWHM (double orange arrow) as indicated. The FWHM values were calculated using $\mathrm{FWHM}=2\sqrt{2\;\ln\;2}\sigma \approx 2.355\sigma$. The FWHM values of the reconstructed image, for both lateral and axial directions, are similar (≈2.5  nm higher) to those of the ground-truth image, indicating the inpainted reconstruction is able to preserve the resolution of a 3D structure. Additionally, we perform the quantitative evaluation of the reconstruction by calculating the root-mean-squared error (RMSE) between the reconstructed image and the ground-truth image and it is shown in Fig. 4. The RMSE values for each reconstruction are the average RMSE values from all the slices. Since the localization list was generated randomly, we conducted 10 simulations and calculated the average RMSE of each reconstruction for the different number of frames. The unit of the RMSE is the same as the intensity (photons) of the image. The curve [Fig. 4] shows significant improvements in the reconstruction with >800 frames, suggesting that increasing frames improve the fidelity of reconstructed structures. The RMSE value for the reconstruction of Fig. 2 using 1000 frames was 0.0748.

**Fig. 5:**
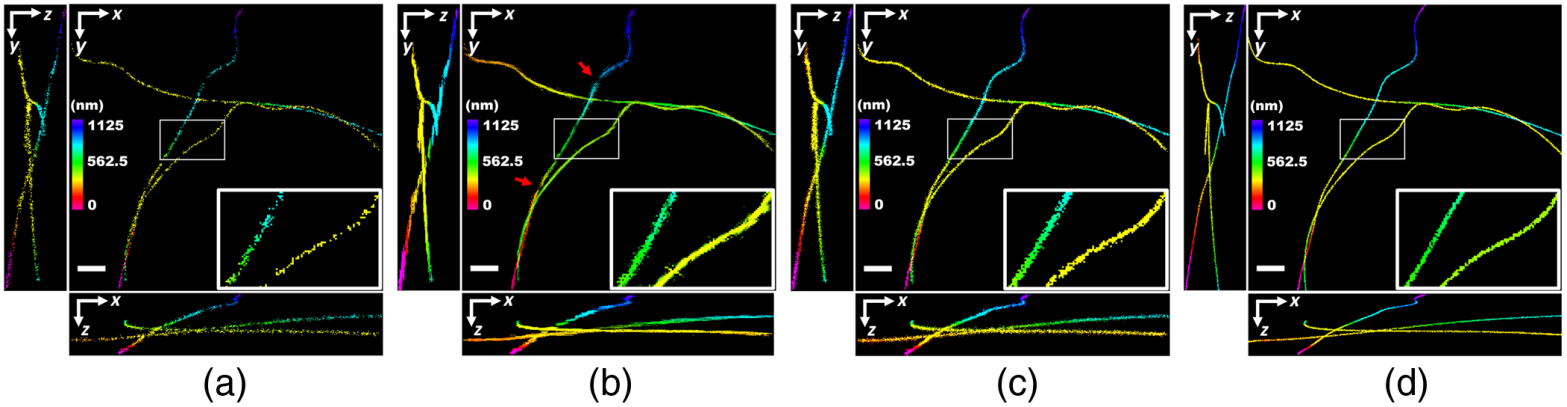
Blind sparse inpainting reconstruction result of realistic simulation data MT0.N1.LD. The (a) low-density; (b) reconstructed; (c) high-density; and (d) the ground-truth super-resolution 3D image with color indicating the depth of z. The low-density image was rendered using 5000 frames and the high-density image was obtained using 19,996 frames. Pixel size: 12.5 nm. Scale bars: 0.5  μm.

In the experimental condition, localization microscopy images are corrupted by noise sources such as false detection from the background noise due to unbound or out of focus light or unspecific binding of antibodies.^40^ To test our method’s performance for realistic simulation conditions, we used publicly available realistic 3D simulation data of microtubules from the École Polytechnique Fédérale de Lausanne (EPFL) 3D SMLM software Benchmarking.^53^ The Alexa 647 labeled STORM data “MT0.N1.LD” consists of 19,996 frames with a molecule density of 0.2 molecules per $\mu m^{2}$. We adopted the 3D-Double Helix datasets and used SMLocalizer^54^ to process diffraction-limited frames. Once the localization list was obtained, we used 5000 frames to generate the low-density 3D image, as shown in Fig. 5(a). To reconstruct the 3D high-density image from the low-density image, we constructed 90 z-slices of the low-density 3D image by grouping the localization data in the z axis with a thickness of 12.5 nm. The field of view (FOV) of the images in Fig. 5 was $5.62\times 5.15\;\mu m^{2}$. The overall axial range was 1.125  μm. Figure 5(b) shows reconstruction using 5000 frames, having much smoother and improved density in both lateral and axial directions. The result is comparable to the high-density image rendered using all frames [Fig. 5(c)]. The ground-truth image is shown in Fig. 5(d). The RMSE values (average of all slices) of the low-density, reconstructed, and high-density images were 0.0167, 0.0144, and 0.0202, respectively. The RMSE values show that our reconstruction has much less deviation from the ground-truth image than the high-density image obtained using 19,996 frames.

### 4.2 Experimental Results

To demonstrate the performance of blind sparse inpainting reconstruction for real 3D SMLM images, we used publicly available localization lists of two microtubules image data and one mitochondrial image data.

**Fig. 6:**
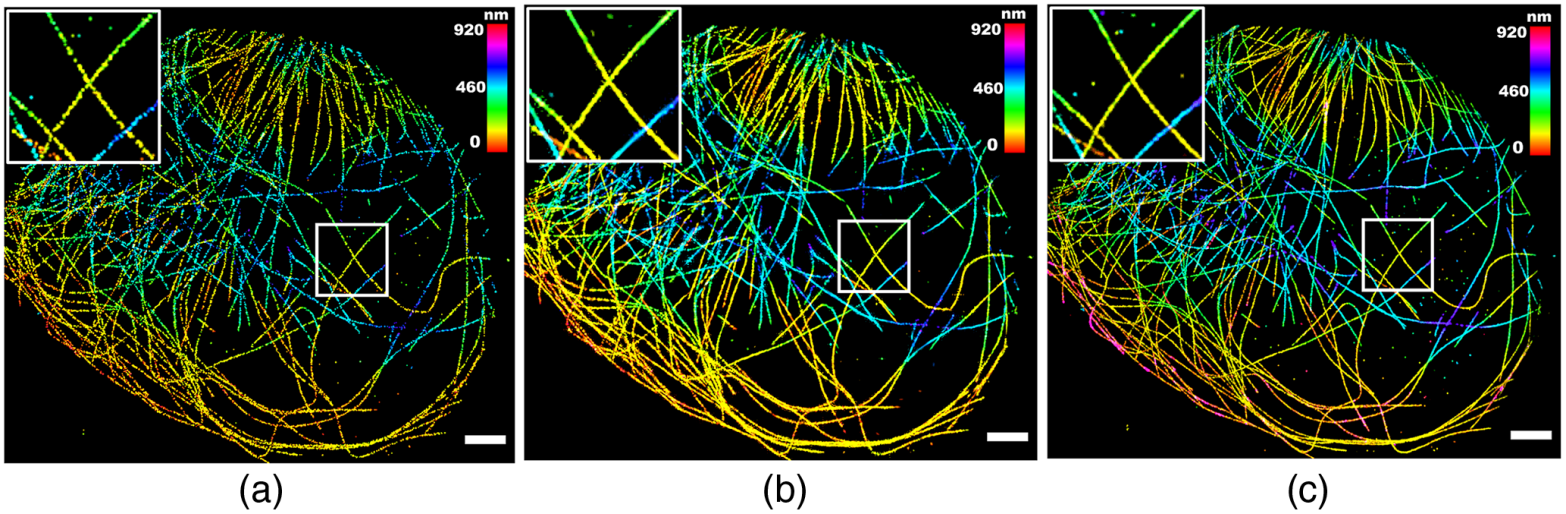
Blind sparse inpainting reconstruction results of Tubulin-A647-3D data. The (a) low-density; (b) reconstructed; and (c) high-density super-resolution 3D image with color indicating the depth of z. The low-density image was rendered using 2254 frames and the high-density image was obtained using 112,683 frames. Pixel size: 40 nm. Scale bars: 3  μm.

**Fig. 7:**
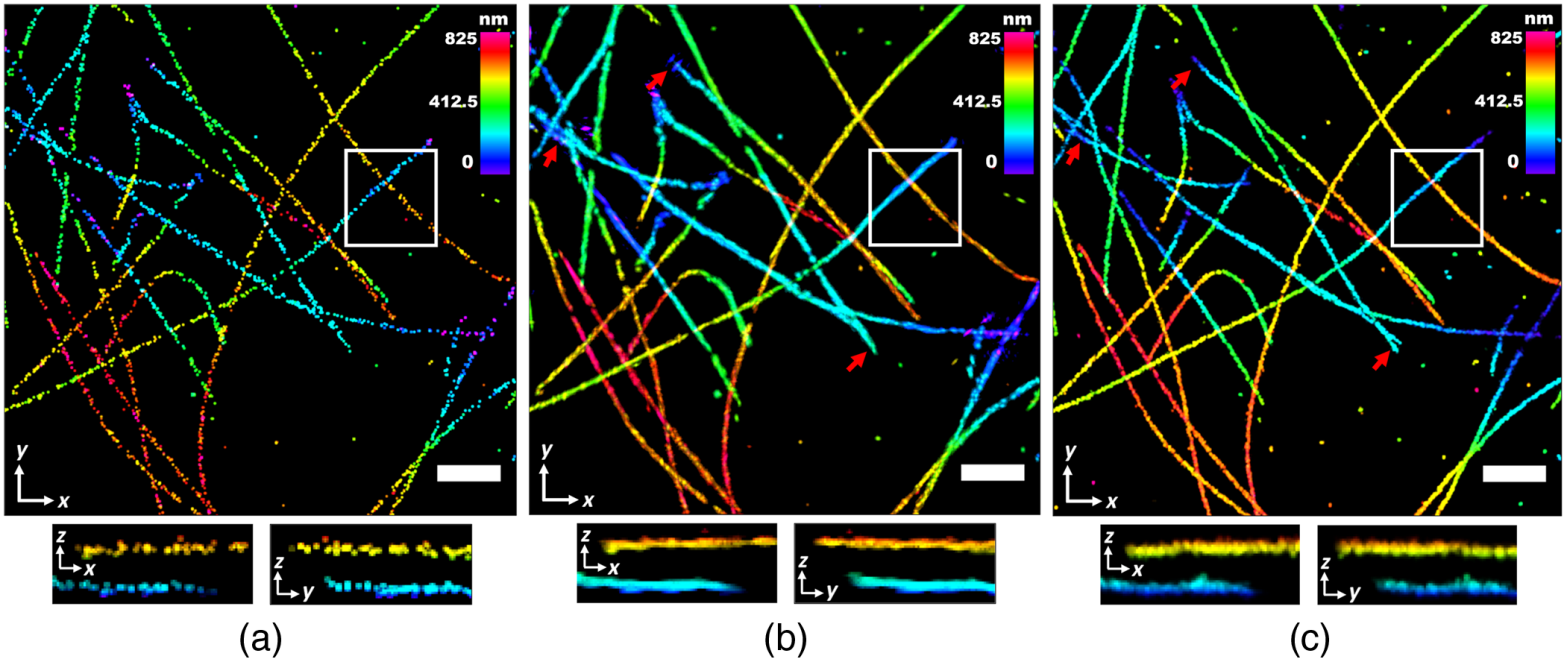
Blind sparse inpainting reconstruction results of an ROI of Tubulin-A647-3D data. The (a) low-density; (b) reconstructed; and (c) high-density super-resolution 3D image with color indicating the depth of z. (x,z) and (y,z) views of the regions enclosed by the white box are also shown. Pixel size: 24 nm. Scale bars: 1.5  μm.

The first data set was from the EPFL SMLM software benchmarking.^53^ The details about sample preparation and microscopy setup of the data can be found in Ref. 55. In brief, microtubules in U-2 OS cells were labeled with anti-alpha tubulin primary and Alexa Fluor 647-coupled secondary antibodies. The diffraction-limited frames (with an exposure time of 15 ms) were imaged using the optical setup of dSTORM with a cylindrical lens. We used the wobble and drift corrected “Tubulin-A647-3D” localization list obtained from 112,683 frames and processed using Super-resolution Microscopy Analysis Platform (SMAP)-2018.^56^ Since the localization list was already available, we did not process the diffraction-limited frame data, but instead directly used them. The isolated localization points due to background noise were filtered using density filtering. When all 112,683 frames with about 1.7 million localization points were used, we obtained a high-density super-resolution 3D image as a reference image [Fig. 6(c)]. The low-density image [Fig. 6(a)] was synthesized using 2254 frames, i.e., 50-fold fewer frames, with about 34 thousand localization points from the same localization list data. To reconstruct the 3D high-density image from the low-density image, we constructed 23 z-slices of the low-density 3D image with FOV of $37.5\times 33.4\;\mu m^{2}$ by grouping the localization list data in z axis with a thickness of 40 nm. The overall axial range was 920 nm. The microtubules filaments could be seen in the low-density image, but structural details were hard to discern. To reconstruct the high-density 3D image, our blind sparse inpainting algorithm was applied to the low-density 3D image. The reconstructed image is shown in Fig. 6(b). The color in Figs. 6(a)–6(c) indicates the depth in the z direction. Visual observation shows that blind sparse inpainting reconstruction significantly improves the localization density of the low-density image. The microtubules filament structures are much denser and more clearly revealed in the reconstruction. Additionally, reconstruction for a region of interest (ROI) ($12\times 12\;{\mu m}^{2}$) of the same data set with much smaller pixel size (24 nm) and z-slice width (Δz=25  nm) is shown in Fig. 7. The superior reconstruction result shows much denser and smoother microtubules structures in both lateral and axial directions.

**Fig. 8:**
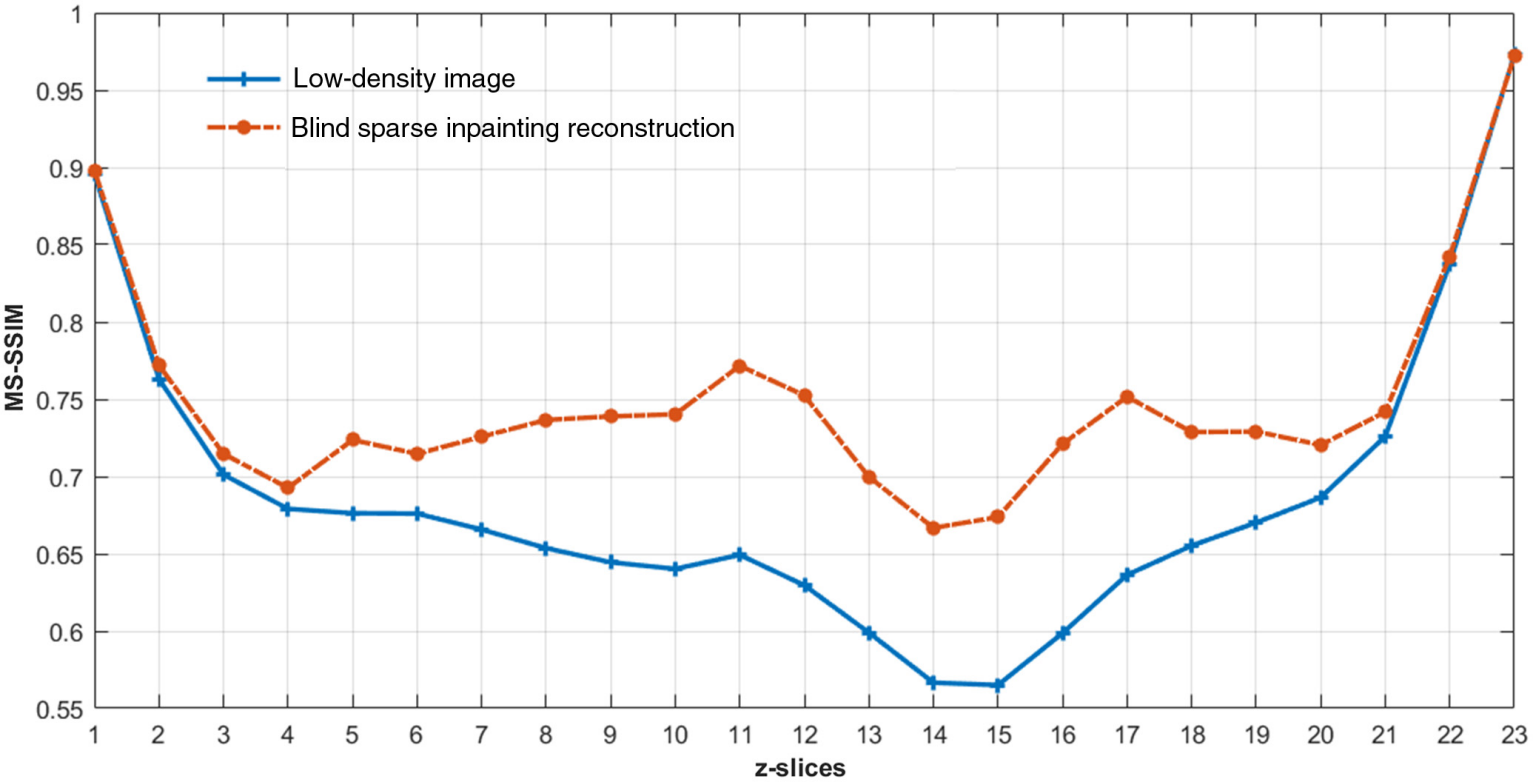
The plot of the MS-SSIM index versus z-slices for comparing the reconstruction of microtubules structures for the Tubulin-A647-3D image of Fig. 6.

For the quantitative evaluation of the reconstructed images of experimental data, we used the multiscale structural similarity index (MS-SSIM),^57^ a perceptually motivated metric, between the reference high-density image and the reconstructed image. Since the ground-truth was not available for the experimental data, the high-density 3D images rendered with all available frames were used as reference images. It is also worth noting that this reference high-density image still might deviate from the ground-truth (as seen in Sec. 4.1). Thus, the RMSE with reference image is not a proper metric for the quantitative evaluation of reconstruction as the pixel value difference can be large even for perfect reconstruction.^42^ Thus, we used MS-SSIM to evaluate the reconstruction capability to capture the structural information along with the slices in the reference image of experimental data sets. The MS-SSIM index has a scale between 0 and 1, with 1 being a perfect match with the reference image. The higher MS-SSIM value indicates a better match of structural information. Figure 8 shows the improvement in the MS-SSIM index of the slices of the reconstructed 3D image [Fig. 6(b)] compared to that of the input low-density 3D image [Fig. 6(a)]. It demonstrates that our method is capable of recovering the structures of microtubules with high similarities to the reference high-density image. The MS-SSIM index of the edge slices (slices 1 to 3, and 21 to 23) are still low because of having very low localization densities with a wide gap between the fluorophore localization in those slices.

**Fig. 9:**
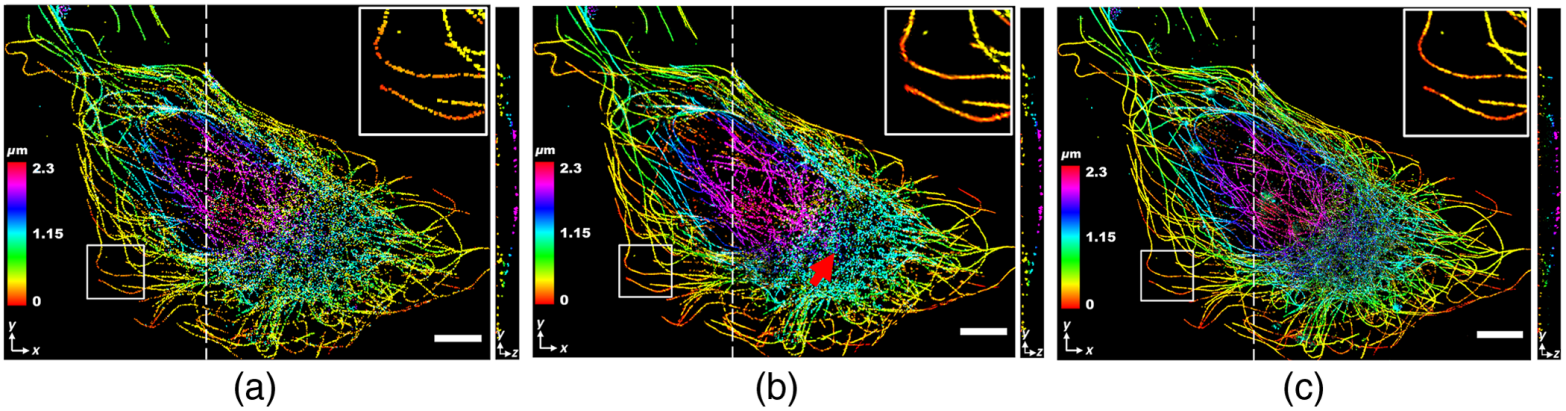
Blind sparse inpainting reconstruction of microtubules data from ZOLA-3D. The (a) low-density; (b) reconstructed; and (c) high-density 3D super-resolution image with color indicating the depth of z. The low-density image was obtained using 4400 frames and the high-density image was obtained using 87,959 frames. The right panel in each image shows a (y,z) slice at the position indicated by the white dashed line. Pixel size: 37 nm. Scale bars: 5  μm.

**Fig. 10:**
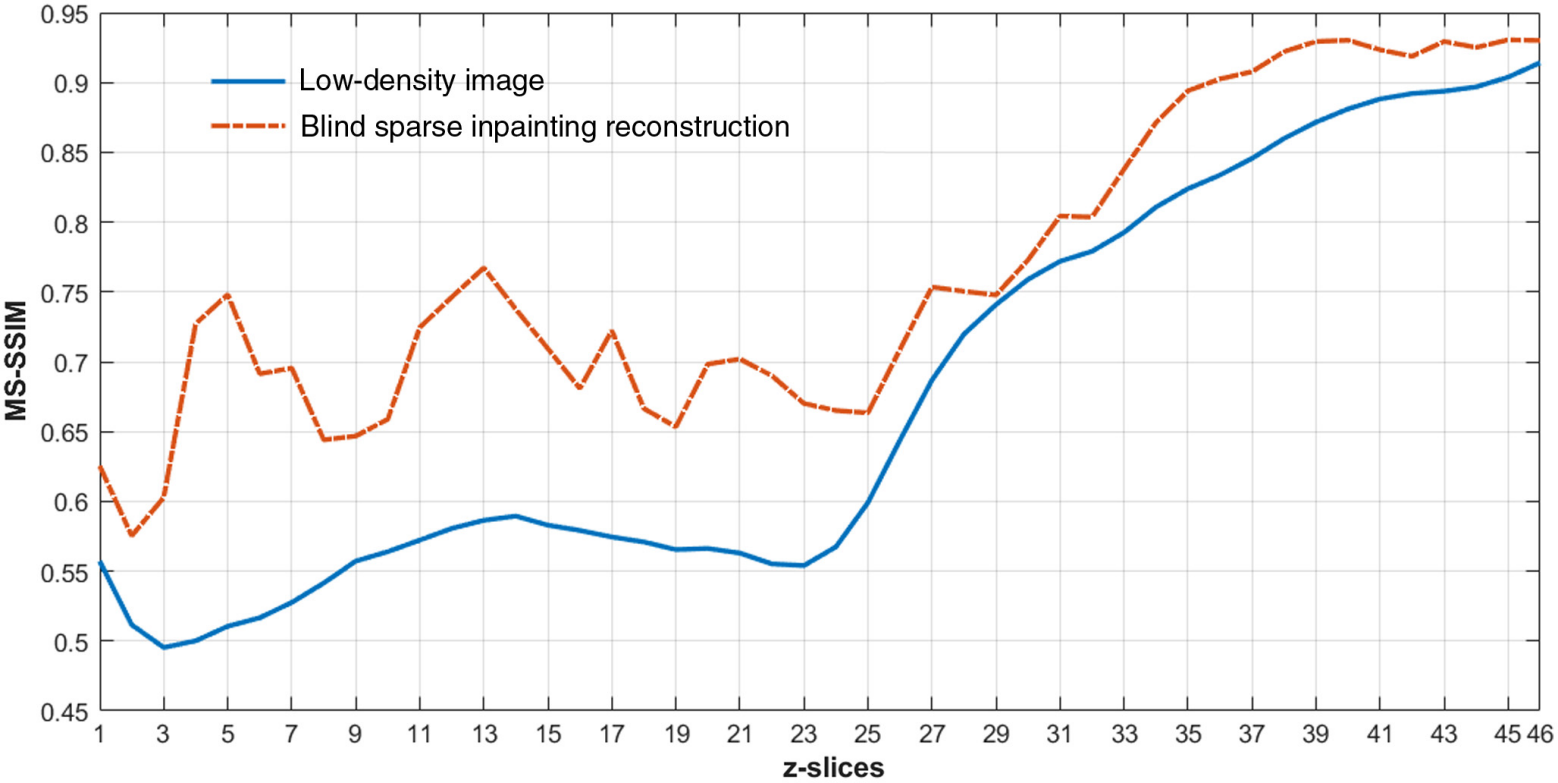
The plot of the MS-SSIM index versus z-slices for comparing the reconstruction of microtubules structures for the data from ZOLA-3D of Fig. 9.

To further evaluate the blind sparse inpainting reconstruction for 3D SMLM experimental data, we used another publicly available microtubule localization list result from Zernike Optimized Localization Approach in 3D (ZOLA-3D).^58^ Details about sample preparation, imaging setup, and processing steps can be found in Ref. 13. In brief, the microtubules in a U-373 MG cell were labeled with anti-alpha tubulin primary and Alexa-647 conjugated secondary antibodies. A total of 87,959 frames were acquired using the saddle point PSF with a variable exposure time of 30 (for the early stage) to 100 ms (in the later stage). Since the localization list was already available, we directly used them. The isolated localization points due to background noise were filtered using density filtering. The high-density 3D super-resolution image [Fig. 9(c)] was generated using all frames with around 899,600 localization points, visualizing the whole cell with an axial range of 2.3  μm. The low-density 3D image [Fig. 9(a)] was generated using 4400 frames, i.e., 20 fold fewer frames, with approximately 57,500 localization points from the same localization data. For reconstruction, we constructed 46 z-slices of the low-density image by grouping the localization data in the z axis with a size of 50 nm with an FOV of $51.58\times 37.62\;{\mu m}^{2}$. Then, the low-density image was given as an input to our blind sparse inpainting algorithm. The reconstructed image is shown in Fig. 9(b). The color in Figs. 9(a)–9(c) indicates the depth in the z direction. Microtubule structures are more clearly revealed in reconstruction with much higher-localization densities comparable to the reference high-density image. Superior reconstructions in the edge of the cell can be observed in the reconstruction. The improvement in the MS-SSIM index, as shown in Fig. 10, also verifies higher similarities with the high-density reference image after the reconstruction. However, some fine features in the high-density image with the dense or close-by structure were not appropriately resolved (red arrow) due to more isolated localization data in those regions.

**Fig. 11:**
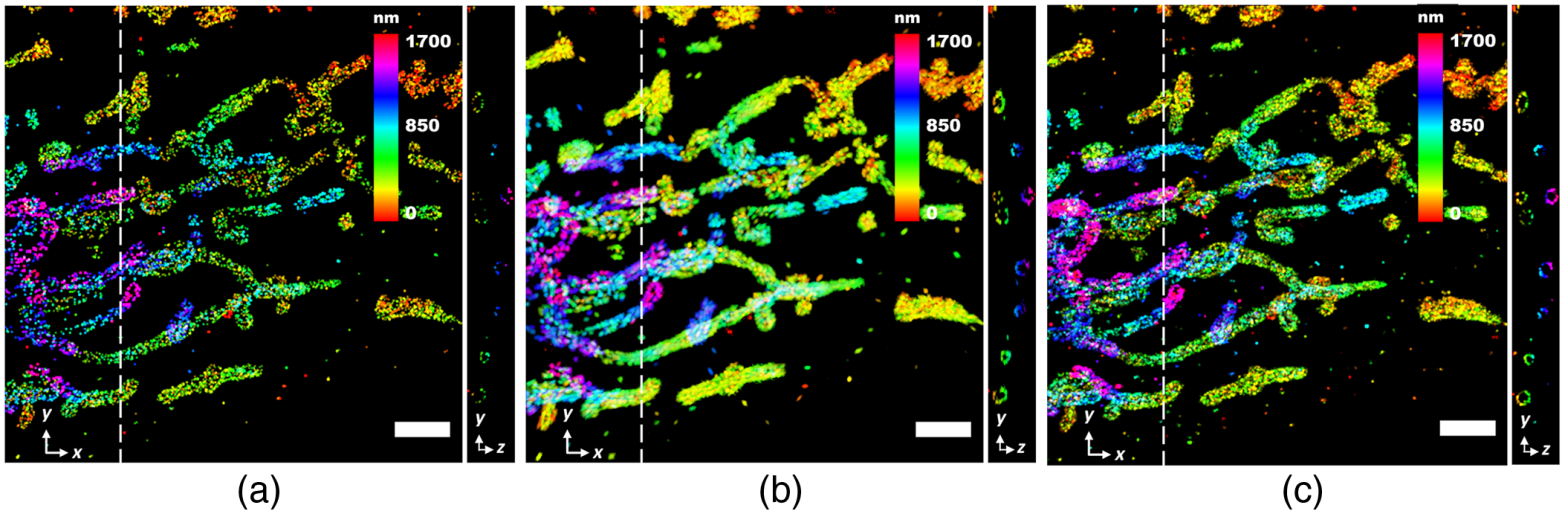
Blind sparse inpainting reconstruction of the mitochondrial 3D image from ZOLA-3D. The (a) low-density; (b) reconstructed; and (c) high-density 3D super-resolution image with color indicating the depth of z. The low-density image was obtained using 5500 frames and the high-density image was obtained using 81,578 frames. The right panel in each image shows a (y,z) slice at the position indicated by the white dashed line. Pixel size: 34 nm. Scale bars: 2  μm.

Similarly, we also evaluate the reconstruction of another 3D SMLM image from ZOLA-3D. The 3D mitochondrial image in COS7 Cells was obtained using saddle point PSF. The high-density 3D image of Fig. 11(c) was rendered using 81,578 frames (≈175,000 localizations after density filtering). For reconstruction, we used 5500 frames (≈19,500 localizations) to generate the low-density 3D image [Fig. 11(a)]. The reconstructed 3D image in Fig. 11(b) shows improvement in the density of the mitochondrial structures both in lateral and axial directions. Due to the tubular structure of the mitochondria, the curvelet transform performed well to give superior reconstruction. The result demonstrates the versatility of our method to reconstruct high-quality 3D super-resolution images by reducing the number of frames.

## 5 Conclusion

We present a computational method based on blind sparse inpainting to reconstruct the high-density 3D images using the low-density 3D images synthesized using very few camera frames obtained from the standard 3D SMLM data. We demonstrate high-quality reconstructions with up to a 10-fold reduction in the number of frames in the simulated 3D SMLM images and up to 50-fold reduction for experimental microtubules 3D SMLM images. Thus, the acquisition time is reduced considerably using fewer camera frames, and the 3D imaging is accelerated without compromising resolution. Furthermore, no change in the existing optical setup or labeling protocol is needed. Additionally, our method can be applied to any of the existing localization algorithms. We expect that our method can offer further improvement in the acquisition time by integrating with the existing higher molecular density labeling methods.

However, the proposed method has several limitations. First, because of the use of the curvelet transform, it may be restricted to the filament structures such as microtubules. For noncurvature structures, appropriate sparsifying transform, such as wavelet transform, can be used. Second, the reconstruction also depends on the localization algorithms. If there are some artefacts due to background noise or incorrect localizations, such artefacts propagate during the reconstructions. Third, when the input image quality is limited due to scarcity of the localization points or increased noise or nonuniform localizations, the reconstructed images may misrepresent the actual structures (e.g., broken structures). Such misrepresentation can be alleviated by improving the input image quality using more frames, but at the cost of reduced acceleration. Finally, since missing localization positions are estimated blindly, there may be some errors in predicting the $P_{Q}$, which may give some artefacts or loss of resolution. Again, such limitations can also be alleviated by using more frames data. We anticipate combining super-resolution optical microscopy and our blind inpainting method will enable future real-time live-cell and high-throughput 3D imaging to investigate the complex nanoscopic biological structures and their functions.
